# Relevance of FXR-p62/SQSTM1 pathway for survival and protection of mouse hepatocytes and liver, especially with steatosis

**DOI:** 10.1186/s12876-016-0568-3

**Published:** 2017-01-13

**Authors:** Sanae Haga, Yimin, Michitaka Ozaki

**Affiliations:** 1 Department of Biological Response and Regulation, Faculty of Health Sciences, Hokkaido University, N-12, W-5, Kita-ku, Sapporo, Hokkaido 060-0812 Japan; 2 Department of Advanced Medicine, Graduate School of Medicine, Hokkaido University, N-15, W-7, Kita-ku, Sapporo, Hokkaido 060-8638 Japan

**Keywords:** FXR, p62/SQSTM1, Nrf2, SHP, Oxidative stress, Liver injury, Steatosis

## Abstract

**Background:**

Liver injury and regeneration involve complicated processes and are affected by various physio-pathological conditions. Surgically, severe liver injury after surgical resection often leads to fatal liver failure, especially with some underlying pathological conditions such as steatosis. Therefore, protection from the injury of hepatocytes and liver is a serious concern in various clinical settings.

**Methods:**

We studied the effects of the farnesoid X receptor (FXR) on cell survival and steatosis in mouse hepatocytes (AML12 mouse liver cells) and investigated their molecular mechanisms. We next studied whether or not FXR improves liver injury, regeneration and steatosis in a mouse model of partial hepatectomy (PH) with steatosis.

**Results:**

An FXR-specific agonist, GW4064, induced expressions of the p62/SQSTM1 gene and protein in AML12 mouse liver cells. Because we previously reported p62/SQSTM1 as a key molecule for antioxidation and cell survival in hepatocytes, we next examined the activation of nuclear factor erythroid 2-related factor-2 (Nrf2) and induction of the antioxidant molecules by GW4064. GW4064 activated Nrf2 and subsequently induced antioxidant molecules (Nrf2, catalase, HO-1, and thioredoxin). We also examined expressions of pro-survival and cell protective molecules associated with p62/SQSTM1. Expectedly, GW4064 induced phosphorylation of Akt, expression of the anti-apoptotic molecules (Bcl-xL and Bcl-2), and reduced harmful hepatic molecules (Fas ligand and Fas). GW4064 promoted hepatocyte survival, which was cancelled by *p62/SQSTM1* siRNA. These findings suggest the potential relevance of the FXR-p62/SQSTM1 pathway for the survival and protection of hepatocytes. Furthermore, GW4064 induced the expression of small heterodimer partners (SHP) and suppressed liver X receptor (LXR)-induced steatosis in hepatocytes, expecting the in vivo protective effect of FXR on liver injury especially with steatosis. In the hepatectomy model of db/db mice with fatty liver, pre-treatment by GW4064 significantly reduced post-PH liver injury (serum levels of LDH, AST & ALT and histological study) and improved steatosis. The key molecules, p62/SQSTM1, Nrf2 and SHP were upregulated in fatty liver tissue by GW4064 treatment.

**Conclusions:**

The present study is the first to demonstrate the relevance of FXR-p62/SQSTM1 and -SHP in the protection against injury of hepatocytes and post-PH liver, especially with steatosis.

**Electronic supplementary material:**

The online version of this article (doi:10.1186/s12876-016-0568-3) contains supplementary material, which is available to authorized users.

## Background

Liver is injured as a result of various physio-pathological events and sequentially regenerates to quantitatively and functionally recover from loss of mass and to compensate for impaired function. Liver has the strong ability to restore lost volume and function, a phenomenon that is rarely seen in other organs [1, 2]. It is well established that normal adult hepatocytes usually are quiescent but have the potential to replicate. After surgical procedures that reduce liver mass/function and induce liver injury, such as partial hepatectomy (PH) or live-donor liver transplantation, a rapid enlargement of the residual or grafted liver commonly occurs to restore the liver mass and function [3]. However, post-PH injury of a diseased liver, for example, in cases of liver cirrhosis or steatosis, or of aged liver, leads to liver failure and is potentially fatal [4–7]. Therefore, a better understanding of the molecular mechanisms of liver injury and protection in various pathological conditions may lead to clinical benefits.

Fatty liver is a commonly encountered hepatic disorder and has various causes, such as obesity, diabetes mellitus, and alcohol consumption [7]. It is often considered a benign condition because it does not usually cause severe clinical symptoms. However, surgical resection of fatty liver and live-donor liver transplantation from a donor with a steatotic liver are problematic because steatosis often causes the remnant liver failure immediately and primary graft non-function [7]. Non-alcoholic steatohepatitis (NASH), has been focused on recently because its clinical importance has become apparent. NASH, characterized by persistent inflammation with mild liver damage, is considered to ultimately result in liver cancer through liver fibrosis and cirrhosis over many years [8, 9].

After hepatectomy, various mitotic factors and cytokines promptly activate various cellular signals and events, eventually leading to sufficient regeneration of the normal liver [1–3, 10, 11]. In the steatotic liver, modified signaling mechanisms due to adaptation to chronic metabolic abnormalities and decreased adenosine triphosphate (ATP) production have been reported as likely causes of increased mortality and impaired regeneration after hepatectomy [7, 12, 13]. Very importantly, hepatic steatosis is considered to reduce tolerance to ischemic injury and oxidative stress (OS) [7, 14].

By the way, p62/SQSTM1 is known as a specific substrate for autophagy and therefore has been used as a marker of autophagy [15, 16]. However, the biological relevance of p62/SQSTM1 was not understood until recently other than autophagy [17–22]. We previously reported that the marked reduction in p62/SQSTM1 in steatotic hepatocytes is a major cause of post-PH liver injury and is possibly involved in acute liver failure following PH [23]. It is known that p62/SQSTM1 directly binds to Keap-1, which inhibits its binding to nuclear factor erythroid 2-related factor-2 (Nrf2) and allows Nrf2 to activate/translocate into the nucleus [18]. Because Nrf2 is a key player in the cellular antioxidant system, it upregulates major antioxidant molecules and also p62/SQSTM1, and protects cells from OS. In addition, it was reported that p62/SQSTM1 phosphorylates/activates Akt, a pro-survival molecule in neuronal cells [21], and reduces the expression of harmful molecules (Fas ligand; FasL and Fas) in liver cells [23]. These facts strongly suggest the pro-survival and anti-cytotoxic effects of p62/SQSTM1 in liver cells.

Nuclear receptors have been widely studied for their clinical relevance in various medical fields. In liver, the farnesoid X receptor (FXR) and liver X receptor (LXR) are deeply involved in glucose and lipid metabolism, and, therefore, are considered to play important physio-pathological roles in homeostasis and survival of living organisms [24–27]. Regarding FXR, it suppresses the sterol regulatory element-binding protein (SREBP)-1c /fatty acid synthase (FAS) pathway through upregulation of small heterodimer partner (SHP) [24, 25, 27]. The SHP negatively regulates the SREBP-1c/FAS pathway and inhibits production of triglycerides (TG) in hepatocytes [27]. Therefore, many clinical trials have been performed expecting the therapeutic efficacy of its agonistic compounds against non-alcoholic fatty liver disease (NAFLD) such as NASH [28]. Recently, there was evidence that FXR directly upregulates the *p62/SQSTM1* gene in hepatocytes [29]. This promptly led us to the idea that FXR activates the pro-survival signals through the upregulation of p62/SQSTM1 and, at the same time, suppresses hepatic steatosis through the upregulation of SHP. If FXR improves hepatic steatosis as well as heals the injury in fatty liver or NASH, the signals of FXR-p62/SQSTM1 may play a pivotal role in maintaining liver homeostasis and protection against injury especially of fatty liver.

In this study, we report that FXR stimulus confers the pro-survival and anti-steatotic properties through induction of p62/SQSTM1 and SHP to hepatocytes, respectively, and that it suppresses post-PH liver injury effectively with reduced fat accumulation in a mouse model. The FXR and p62/SQSTM1-mediated signals of hepatocytes seem to be relevant in surviving and protecting hepatocytes in various liver conditions especially with fatty change.

## Methods

### Cell culture, reagents, and siRNAs

The alpha mouse liver 12 (AML12) cells, established from hepatocytes from a mouse transgenic for human transforming growth factor-α (TGF-α), express high levels of human TGFα and lower levels of mouse TGFα (ATCC, Manassas, VA, USA). AML12 cells were maintained at 37 °C in 5.0% CO_2_ in Dulbecco’s Modified Eagle Medium: Nutrient Mixture F-12 (DMEM/F12) (Gibco, CA, USA) supplemented with 10% fetal bovine serum. GW4064, an agonist of FXR, and T0901317, an agonist for LXR-α, were purchased from Sigma-Aldrich Co., LLC. (St. Louis, MO, USA) and Merck Millipore Corporation (Darmstadt, Germany), respectively. Small interfering RNAs (siRNAs) for mouse *p62/SQSTM1* (sense *5′-GGAACUCGCUAUAAGUGCATT-3′*, antisense *5′-UGCACUUAUAGCGAGUUCCCA*) and *GAPDH* used as the control were purchased from Ambion, Inc. (Austin, TX, USA). Transfection of siRNAs into AML12 liver cells was accomplished using Lipofectamine 2000 (Invitrogen, Rockville, MD, USA) according to the manufacturer’s instructions. p62/SQSTM1 and GAPDH expressions were both evaluated by PCR and Western blot analyses.

### Reverse transcription-PCR assay

First-strand cDNA synthesis used 2.5 μg of total RNA from AML12 cells, Superscript III reverse transcriptase, and oligo(dT)_20_ primers (Invitrogen, Carlsbad, CA, USA), according to the manufacturer’s instructions. The cDNA was amplified by PCR with specific primers for mouse p62/SQSTM1 (225 bp): sense 5′-GATGTGGAACATGGAGGGAAGAG-3′, antisense 5′-AGTCATCGT CTCCTCCTGAGCA-3′. PCR was performed by 27 cycles of denaturation at 94 °C for 30 s, annealing at 55 °C for 30 s, and extension at 72 °C for 30 s.

### Monitoring and evaluation of cell survival, cell death and liver injury

Cells at 40–50% confluence were plated in a plate. Cell survival was determined by plating the cells in the xCELLigence System (Roche, Basel, Switzerland), which allows for automated non-invasive, real-time, and label-free monitoring of live cells in culture. For evaluation of cell death, we examined lactate dehydrogenase (LDH) release from hepatocytes into culture media. “LDH cytotoxicity detection kit” (Takara, Otsu, Japan) was used according to the manufacturer’s instructions. Briefly, the LDH reaction mixture was added to the aliquot taken from the media for cell culture 72 h after the treatment with GW4064 and incubated at room temperature for 30 min. The absorbance at 490 nm was measured using a multi-well plate reader. In mouse experiments, biochemical analyses, such as for serum levels of aspartate aminotransferase (AST), alanine aminotransferase (ALT), and LDH were performed as indices of liver injury before and after PH.

### Adipogenesis assay

Adipogenesis was evaluated by an “adipogenesis colorimetric/fluorometric assay kit” (BioVision, Milpitas, CA, USA) according to the manufacturer’s instructions. Briefly, cultured cells or liver tissues were harvested, washed by phosphate buffered saline (PBS), and stored at −80 °C before the assay. The specimens were completely dissolved by the lipid extraction buffer provided by the manufacturer. For the TG assay, 5–50 μl of the lipid extracts was transferred to a 96-well plate and the volume was brought to 50 μl with the assay buffer. Specimens and standards were added with 2 μl of lipase, mixed, and incubated 10 min at room temperature to convert TG to glycerol and fatty acid. The samples and standards were mixed with 50 μl of the reaction mix and measured at 570 nm for the colorimetric assay. Nile red stain was used to quantify intracellular lipid accumulation in cultured cells. T0901317- and GW4064-treated AML12 cells were rinsed with PBS and stained with the lipid-specific Nile red stain (AdipoRed Assay Reagent, Lonza, Basel, Switzerland). After the incubation at room temperature for 10 min, cells were applied for the fluorescence assay with excitation at 485 nm and emission at 572 nm (expressed as relative fluorescence units, RFU).

### Western blot analysis

Western blot analysis was performed with appropriate antibodies specific for Nrf2 (1:200 dilution), heme oxygenase-1 (HO-1, 1:500 dilution), manganese-dependent superoxide dismutase (Mn-SOD, 1:1000 dilution), thioredoxin (TRX, 1:500 dilution) (BD Transduction Laboratories, NJ, USA), Bcl-2 (1:200 dilution), Bcl-xL (1:200 dilution), Fas (1:200 dilution), SHP (1:100 dilution) (Santa Cruz Biotechnology Inc., Santa Cruz, CA, USA), FasL (1:200 dilution) (Abcam, Cambridge, UK), catalase (1:1000 dilution) (EMD Biosciences, Darmstadt, Germany), p62/SQSTM1 (1:1000 dilution), phospho-Akt (1:1000 dilution), and Akt (1:1000 dilution) (Cell Signaling Technology Inc., Danvers, MA, USA) . Whole cell or tissue protein extracts (25 μg) were separated by 10% sodium dodecyl sulfate-poly acrylamide gel electrophoresis (SDS-PAGE) and transferred to a polyvinylidene difluoride (PVDF) membrane. After blocking in 5% skim milk-PBS with 1% Tween 20 (PBS-T), the membrane was incubated in the primary antibody diluted properly (as indicated above) in PBS-T buffer containing 2% bovine serum albumin (BSA) for overnight at 4 °C, and washed 3 times in PBS-T. The membrane was next incubated with anti-mouse or anti-rabbit secondary antibody conjugated to horseradish peroxidase (HRP) (1:5000 dilution) in a blocking buffer (5% skim milk in PBS-T) at room temperature for 1 h. The membrane was finally applied to chemiluminescent HRP detection reagent (Luminata Forte Western HRP substrate, Merck Millipore, Darmstadt, Germany) and the chemiluminescent signals were detected using a CCD imaging system (LuminoGraph, ATTO, Tokyo, Japan).

### Activation assay of Nrf2 in AML12 cells

Activation of Nrf2 in AML12 cells was evaluated by immunofluorescence microscopic observation. Cells cultured on a glass bottom dish were stimulated by GW4064 and then fixed with ice-chilled methanol for 5 min and permeabilized with 0.1% Triton X-100 in PBS for 10 min at room temperature. After blocking treatment, cells were labeled with anti-Nrf2 as a primary antibody for 1 h at room temperature, followed by incubation with a secondary antibody conjugated Alexa Fluor 488 (Thermo Fisher Scientific Inc., Waltham, MA, USA). Then nuclei were counterstained with Hoechst 33342. We investigated the expression and localization of Nrf2 in AML12 cells with a fluorescent microscope (Biozero; Keyence Corp., Osaka, Japan).

### Animal experiments

Male homozygous leptin receptor-deficient (db/db) mice (45–50 g body weight, 12 weeks old) were obtained from CLEA Japan (Tokyo, Japan) and used for the 2/3 PH experiment. GW4064 was administered daily intraperitoneally (5 mg/kg body weight) for 5 days (3 times before and 2 times after the surgical procedure). The mice were fasted overnight prior to the experiments and were anesthetized with 1.5–2.0% isoflurane (Forane©, Abbott, Tokyo, Japan). The laparotomy was performed by mid-abdominal incision and the median and left liver lobes were exposed. After ligating the vessels to each liver lobe with 3–0 braided silk at the base of each lobe, the median and left liver lobes were surgically resected. The mice underwent laparotomy after anesthesia, and were closed without liver lobe ligation and resection for sham operation. Mice were sacrificed for the collection of liver specimens at the indicated time points before and after hepatectomy, and the liver/body weight ratios were calculated to estimate the recovery of liver mass. The percentage of the whole liver constituted by each lobe was similar to the lean mice, and surgical resection of the middle and left liver lobes resulted in 2/3 PH. Sudan III stained lipid droplets in more than 90% of the hepatocytes in the liver of db/db mice. The animals were maintained under standard conditions and treated according to the Guidelines for the Care and Use of Laboratory Animals of Hokkaido University.

### Histological analysis

Liver tissues were fixed in 10% buffered formalin, paraffin embedded, and subjected to hematoxylin and eosin staining (H & E). To visualize lipid accumulation in the liver, frozen sections of formalin-fixed liver tissue were stained with Sudan III. Briefly, the liver frozen sections (8 μm thick) were prepared on slide glasses, air-dried and rinsed with 50% ethanol. Next, the specimens were stained in Sudan III stain for 10 min at room temperature, and rinsed again to remove excess stain. Counterstain (nuclear stain) was performed with hematoxylin stain for 3 min. After washing gently several times by water, the specimens were mounted with coverslip and microscopically observed.

### Statistical analysis

All results were expressed as means ± standard error of the mean (SEM). Data were compared by Fisher’s test, and *p* values of less than 0.05 were considered to be statistically significant.

## Results

### GW4064, a specific agonist of FXR, induced p62/SQSTM1 in AML12 liver cells

**Fig. 1:**
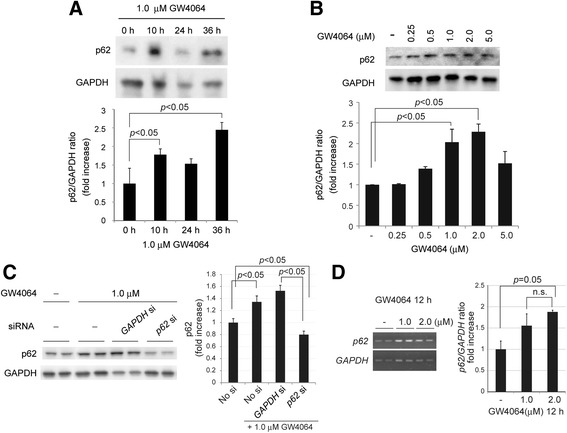
FXR-agonist induced p62/SQSTM1 in AML12 liver cells. **a** GW4064 (1.0 μM), an FXR-specific agonist, expressed p62/SQSTM1 protein 10 h after administration and continued at least 36 h in AML12 liver cells. The immunoblot is a representative of the three independent experiments. **b** p62/SQSTM1 protein was expressed by GW4064 in a dose-dependent manner (0.25–5.0 μM) at 36 h after the treatment. **c** The protein induction of p62/SQSTM1 by GW4064 (1.0 μM) was cancelled by *p62/SQSTM1* siRNA (10 nM). **d** Reverse transcription–PCR analysis of the *p62/SQSTM1* gene in AML12 liver cells. GW4064 robustly upregulated the *p62/SQSTM1* gene in 1.0 and 2.0 μM of GW4064. *Each blot* represents at least three independent experiments (**a**, **b**, **c**). ImageJ software was used for quantitative analysis of western blot and reverse transcription–PCR. Data are expressed as mean ± SEM. *p* values <0.05 were considered statistically significant

We first attempted to confirm the expression of p62/SQSTM1 in AML12 liver cells by FXR stimulus. The expression of p62/SQSTM1 protein was observed 10 h after treatment with a specific agonist of FXR, GW4064 (1.0 μM) (Fig. 1a), and continued for at least 36 h. The protein expression of p62/SQSTM1 at 36 h after the treatment with GW4064 was observed in a dose-dependent manner in a range of 0.25 to 2.0 μM (Fig. 1b). The protein induction of p62/SQSTM1 by GW4064 (1.0 μM) was significantly reduced by siRNA of the *p62/SQSTM1 gene* (Fig. 1c). Furthermore, GW4064 induced the mRNA expression of *p62/SQSTM1* in AML12 liver cells (Fig. 1d).

These data indicate that FXR-agonist upregulates *p62/SQSTM1* gene expression and induces transcriptionally its protein expression in non-tumorous AML12 liver cells, supporting the previously reported data that the *p62/SQSTM1* gene is upregulated by FXR in HepG2 cells [29].

### An FXR agonist sent signals to anti-oxidant and pro-survival molecules but not mitosis-associated molecules in AML12 liver cells

**Fig. 2:**
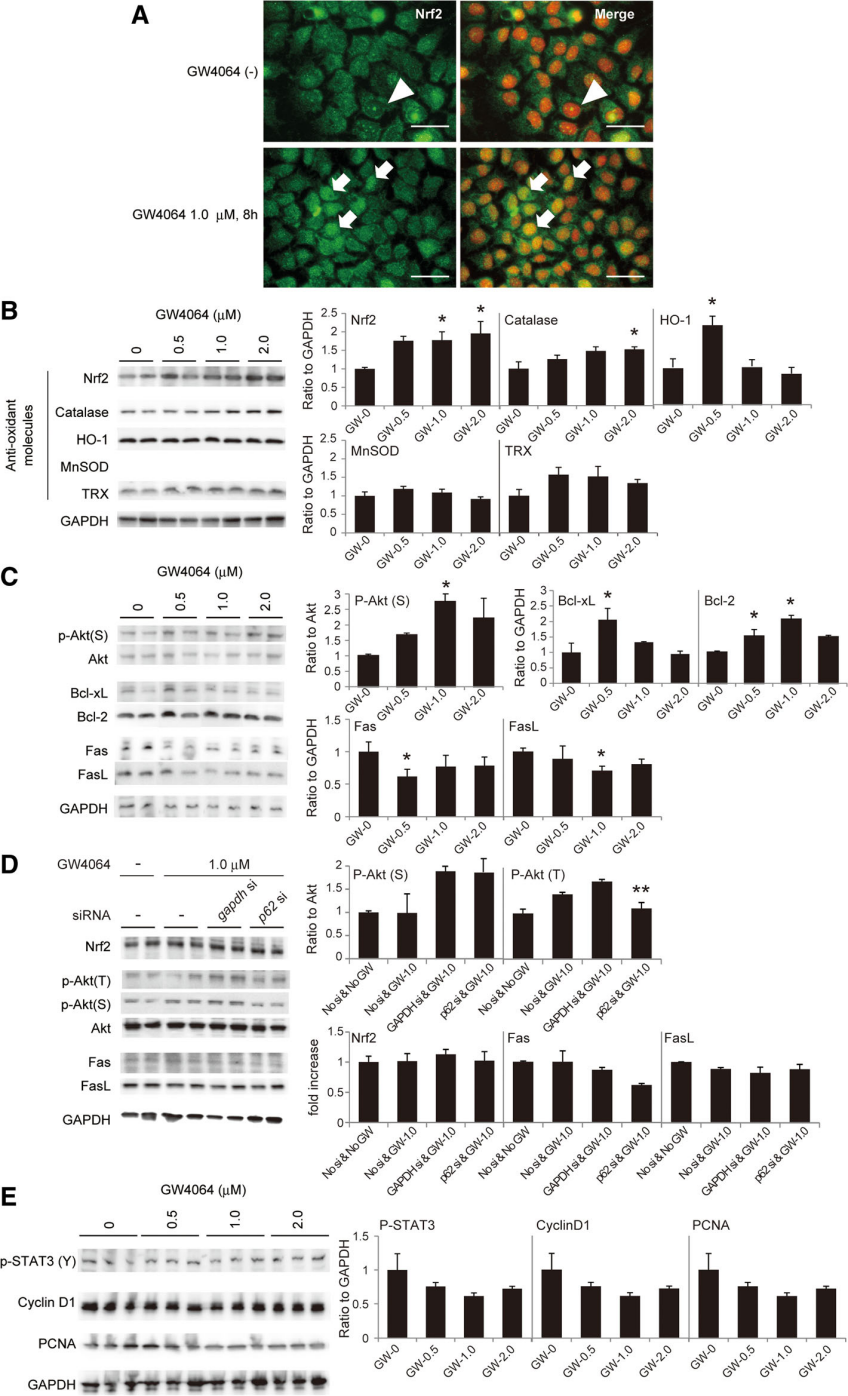
FXR-agonist activated Nrf2 and induced the expressions of Nrf2 & anti-oxidant molecules in AML12 cells. **a** Nrf2 in cytosols was translocated into nuclei by GW4064 treatment (1.0 μM) in AML12 cells. Nrf2 was not stained in nuclei without GW4064 (*arrowheads*), but clearly stained in nuclei 8 h after GW4064 administration (*arrows*). 4′ 6-diamidino-2-phenylindole dihydrochloride (DAPI) was used for nuclear stain in blue (*pseudo-colored red*). Scale bar, 50 μm **b** The protein expression of Nrf2 and the antioxidant molecules (catalase, heme oxygenase-1; HO-1, manganese-dependent superoxide dismutase; MnSOD, and thioredoxin; TRX) 36 h after the treatment with GW4064 (1.0 μM). **c** GW4064 induced phosphorylation of Akt and anti-apoptotic molecules (Bcl-xL and Bcl-2), and suppressed the expressions of harmful hepatic molecules such as FasL and Fas in AML12 liver cells. **d** Ablation of GW4064 by *p62/SQSTM1* siRNA reduced phosphorylation of Akt, which was induced by GW4064 in AML12 liver cells, though this does not affect the protein expressions of the other molecules. **e** GW4064 did not affect cell cycle-associated molecules. GW4064 did not phosphorylate STAT3 (Y705) nor induce cyclinD1 and PCNA. Each experiment was performed three times and representative photographs are shown. The duplicates of immunoblots are taken from the specimens of experiments performed at different times. ImageJ software was used for quantitative analysis of western blot. The quantitative analysis data are expressed as mean ± SEM. *p* values <0.05 were considered statistically significant (*: *p* < 0.05 vs GW-0 μM group; **: *p* < 0.05 vs *GAPDH* siRNA & GW-1.0 μM group)

Because FXR induced p62/SQSTM1 in AML12 cells (Fig. 1), we next studied the effect of the FXR-agonist on the expression of Nrf2 and its nuclear translocation (activation), and also the expression of the Nrf2-associated anti-oxidant molecules in AML12 cells. Treatment with GW4064 (1.0 μM) induced the rapid and marked translocation of Nrf2 into nuclei of AML12 liver cells (Fig. 2a). The immunocytochemical study clearly showed that the nuclear translocation of Nrf2 occurred within 8 h after the treatment with GW4064 and continued in nuclei at least 36 h. Interestingly, the nuclear translocation of Nrf2 was most evident at 1.0 μM (Additional file 1), though the protein was expressed more strongly at 2.0 μM (Fig. 2b). These findings indicate that GW4064 even at lower concentrations rapidly and persistently induces Nrf2 activation in AML12 liver cells. As expected, GW4064 induced significant expression of Nrf2-associated anti-oxidant-associated molecules such as Nrf2, catalase and HO-1 36 h after the treatment in AML12 liver cells (Fig. 2b). Mn-SOD and TRX were mildly induced, but not significantly. Interestingly, HO-1 was induced evidently at the low concentration of GW4064 (0.5 μM), different from other molecules.

Next, we studied the pro-survival effects of FXR on AML12 liver cells. GW4064 induced the phosphorylation of Akt (Fig. 2c). GW4064 also induced anti-apoptotic proteins such as Bcl-xL and Bcl-2 significantly at 0.5 and 1.0 μM. respectively. The protein expressions of Fas and FasL, which induce harmful effects on hepatocytes, were also evidently suppressed by GW4064 administration. Though deletion of p62/SQSTM1 clearly reduced FXR-induced phosphorylation of Akt (T308), this did not affect the protein expression of the pro-survival- and apoptosis-associated molecules examined in this study (Fig. 2d). These facts indicate that these critical molecules for cell survival and apoptosis are not only regulated by FXR-p62/SQSTM1 pathway, but also by the mechanisms other than FXR-p62/SQSTM1.

Regarding cell cycle-associated molecules, the treatment with GW4064 did not affect phosphorylation of the signal transducer and activator of transcription 3 (STAT3) (Y705) nor protein expressions of cyclinD1 and proliferating cell nuclear antigen (PCNA) (Fig. 2e).

### Activation of the FXR-p62/SQSTM1 pathway improved liver cell survival

**Fig. 3:**
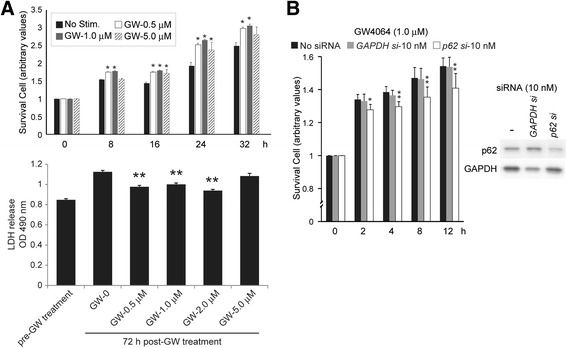
FXR-agonist improved cell survival through p62/SQSTM1 in AML12 liver cells. **a** GW4064 improved cell survival significantly at 0.5, 1.0 and 5.0 μM, showing the peak effect at 1.0 μM. (*: *p < 0.05* vs non-stimulant group) GW4064 significantly suppressed LDH release 72 h after the treatment at 0.5, 1.0 and 5.0 μM. (**: *p < 0.05* vs GW-0 μM group) **b** Cell survival effects of GW4064 was cancelled partially but significantly by p62/SQSTM1 knockdown (10nM of siRNA of *p62/SQSTM1* gene). (* : *p < 0.05* vs no siRNA group; **: *p < 0.05* vs no siRNA & *GAPDH* siRNA groups) Each experimental group consisted of at least three independent experiments. Data are expressed as mean ± SEM. *p* values <0.05 were considered statistically significant

We thereafter studied the effect of the FXR-agonist on liver cell survival and cell death by monitoring live liver cells chronologically and LDH release, respectively (Fig. 3a). We studied the pro-survival effect of GW4064 at concentrations of 0 to 5.0 μM. GW4064 prolonged cell survival significantly in 0.5–1.0 μM, showing the peak effects at 1.0 μM. In the same range of concentrations (0.5–2.0 μM), GW4064 reduced mildly but significantly LDH release (Fig. 3a). Interestingly, the most effective concentration of GW4064 for cell survival (1.0 μM) was almost coincident to that of the protein expression of p62/SQSTM1 (Fig. 1b). These facts may indicate that the pro-survival effect of GW4064 mainly by protection against cell injury. This effect of GW4064 was significantly reduced by silencing the *p62/SQSTM1* gene (Fig. 3b), indicating the possible involvement of FXR in cell survival/cell protection via FXR-p62/SQSTM1-associated pathway.

### FXR-agonist showed adipogenic and adipolytic effects in hepatocytes, and protected steatotic hepatocytes from injury

**Fig. 4:**
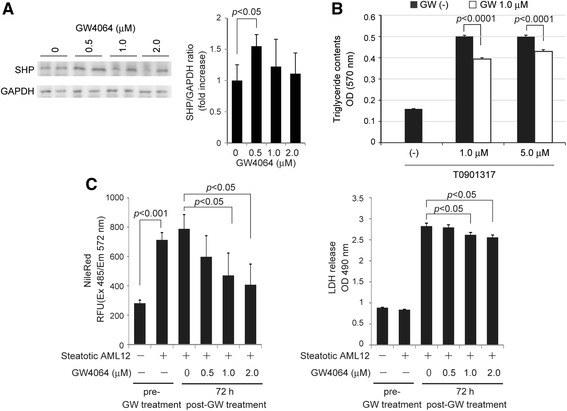
FXR-agonist induced expression of SHP, showed adipogenic and adipolytic effects in hepatocytes, and protected steatotic hepatocytes. **a** Expression of SHP was induced by GW4064 (0.5 to 2.0 μM) 36 h after the treatment in AML12 liver cells. *Each blot* represents at least three independent experiments. The duplicates of immunoblots are taken from the specimens of experiments performed at different times. ImageJ software was used for quantitative analysis of western blot. **b** Steatosis was observed in AML12 liver cells by treatment with 1.0 and 5.0 μM of T0901317 (a specific agonist of LXR) for 7 days. The accumulation of TG induced by T0901317 was significantly suppressed by the concurrently administered GW4064 (1.0 μM). **c** The adipolytic and protective effects of GW4064 were evaluated by using LXR-induced steatotic hepatocytes. The treatment with T0901317 robustly reduced TG contents in a dose-dependent manner, and reduced cell death (LDH release) of the steatotic hepatocytes. Each experiment was performed three times and the data are expressed as mean ± SEM. *p* values of <0.05 were considered statistically significant

We studied the expression of SHP by GW4064 at the same concentrations where GW4064 showed pro-survival effects in AML12 liver cells (Fig. 4a). Though SHP was slightly expressed without GW4064 administration, its protein expression was upregulated by GW4064 similarly at 0.5 to 2.0 μM (Fig. 4a). So, we confirmed whether GW4064 inhibits LXR-induced TG accumulation in AML12 liver cells.

GW4064 (1.0 μM and 5.0 μM), when concurrently administered with T0901317 (an LXR-agonist), effectively suppressed TG accumulation in hepatocytes (Fig. 4b). We also examined whether or not GW4064 reduces the accumulated TG in hepatocytes after the treatment with T0901317 (Fig. 4c). GW4064 reduced TG content of AML12 liver cells dose-dependently, showing the adipolytic effect on steatotic hepatocytes. In the steatotic hepatocytes, GW4064 reduced mildly but significantly LDH release from steatotic hepatocytes (cell protective effect). Though the protective effect of hepatocytes by GW4064 was not great, it showed evident adipolytic effect in the steatotic hepatocytes. These facts led us to expect the in vivo effect in a mouse model with fatty liver.

### LXR-agonist improved post-PH liver injury and steatosis in db/db mice

**Fig. 5:**
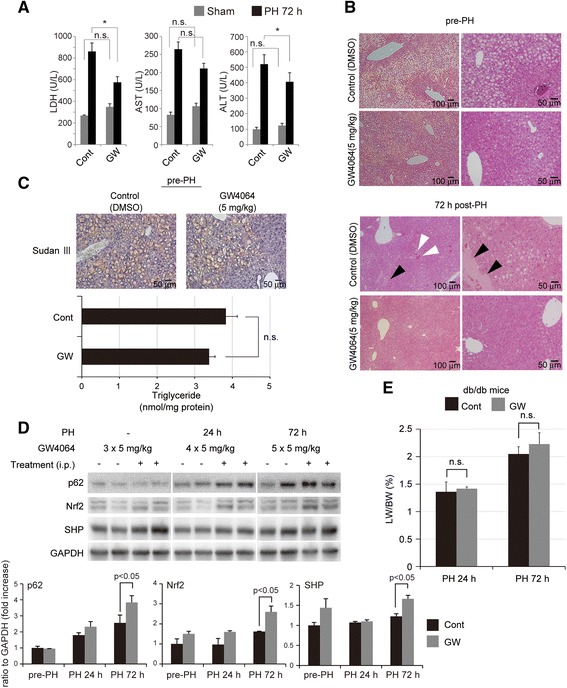
FXR-agonist improved post-PH liver injury and steatosis in db/db mice. **a** Blood biochemistry data are shown. Serum levels of LDH, AST and ALT were reduced 72 h post-PH by the treatment with GW4064 (5 mg/kg BW, refer to Materials and Methods for details). **b** Hematoxylin and eosin (H & E) staining of liver tissue. *Upper panel*: The droplets of hepatocytes were obviously reduced in the GW4064-treated mice before PH. *Lower panel*: The detachment of endothelial cells (*arrowhead in black*) and spotty necrosis with hemorrhage (*arrowheads in white*) observed 72 h post-PH in control mice were not notable in the GW4064-treated mice. **c** Sudan III staining of liver tissue revealed that steatotic hepatocytes were obviously reduced in the GW4064-treated mice without PH. The content of TG was reduced in the liver treated with GW4064, but not statistically significant. **d** Western blot analysis revealed the increased expression of p62/SQSTM1, Nrf2 and SHP in the liver tissue of the GW4064-treated mice. *Each blot* represents at least three independent experiments. The duplicates of immunoblots are taken from the specimens of experiments performed at different times. ImageJ software was used for quantitative analysis of western blot. **e** Post-PH liver mass recovery was slightly improved in the GW4064-treated mice, but the difference was not statistically significant. At least four mice were used for each experiment and the representative data are shown (**a**, **b**, **c**, **e**). The data are expressed as mean ± SEM (**a**, **c**, **d**, **e**). *p* values <0.05 were considered statistically significant

Lastly, we examined in vivo effects of FXR by using a mouse PH model with fatty liver. We studied whether a FXR-agonist suppresses post-PH liver injury, improves steatosis and recovery in the db/db mouse. To study the injury of steatotic liver, we evaluated biochemical markers (serum levels of LDH, AST, and ALT) and histological changes. A significant improvement in liver injury was observed in the GW4064-treated mice 72 h post-PH (Fig. 5a and b). The biochemical markers in both groups (GW4064-untreated and -treated groups) similarly increased immediately after PH (24 h post-PH), possibly due to the direct mechanical injury to the liver by surgical maneuver. However, an improvement in the biochemical markers was observed in the GW4064-treated mice 72 h post-PH (Fig. 5a). Histological examination also supported the blood biochemistry data, showing that detachment of endothelial cells of central veins (arrowheads in black) and spotty cellular necrosis and hemorrhage (arrowheads in white) were observed mainly in the liver of non-treated db/db mice, but not in the liver of the GW4064-treated mice (Fig. 5b). The mitotic hepatocytes were not particularly observed both in GW4064-treated and non-treated livers. These data and observations indicate that the treatment with GW4064 (5 mg/kg BW) suppressed post-PH liver injury, but did not affect mitotic response. Histological examination (H & E and Sudan III stains) also revealed that lipid accumulation in liver was obviously lower pre- and post-PH in the GW4064-treated mice (Fig. 5b and c). Biochemical analysis of TG contents in liver tissue also showed reduction tendency of TG in the livers of GW4064-treated mice (Fig. 5c), though the difference was not statistically significant. Serum levels of glucose and TG after PH were not affected by GW4064 treatment (Additional file 2).

Western blot analysis revealed that GW4064 induced p62/SQSTM1, Nrf2 and SHP significantly after PH (Fig. 5d). SHP protein was induced even before PH (pre-operative 3 day-administration of GW4064, 5 mg/kg) continued until 72 h post-PH. Post-PH liver mass recovery was improved slightly 24 h post-PH and more 72 h post-PH by GW4064 treatment, but the difference was not statistically significant (Fig. 5e).

## Discussion

Recently, p62/SQSTM1 has been considered to play major roles in the protection from injury in various organs and pathological conditions, independent of autophagy [18–23]. In liver, we previously demonstrated that steatosis induced the reduction of p62/SQSTM1 in hepatocytes, which causes post-PH necrotic and apoptotic acute liver injury by enhancing OS and the FasL/Fas signaling pathway [23]. p62/SQSTM1 positively regulated the DNA binding activity of Nrf2 by physical association with Keap-1 and activates its transcriptional activity on *ARE*. Therefore, reduction of p62/SQSTM1 in hepatocytes, regardless of the existence of steatosis, lowered the expression of anti-oxidant molecules (catalase, MnSOD, Ref-1, HO-1, TRX, and GPx) by increasing Keap-1/Nrf2 binding (i.e., decreasing *ARE* activity), making the hepatocytes/liver susceptible to harmful OS. The increases in FasL and Fas by reduced p62/SQSTM1 led to caspase-mediated apoptosis and OS-mediated necrosis in steatotic hepatocytes/liver. These mechanisms were likely to be a main cause of post-PH fatty liver injury. On the contrary, overexpression of p62/SQSTM1 by gene transduction increased the expressions of anti-oxidant molecules through activation of the Nrf2 pathway and also reduced harmful hepatic molecules such as FasL and Fas in hepatocytes and liver. Therefore, upregulation of p62/SQSTM1 seems to be a good clinical target for liver protection, especially against fatty liver and NASH where OS and FasL/Fas undoubtedly contribute to fatty liver injury [23].

**Fig. 6:**
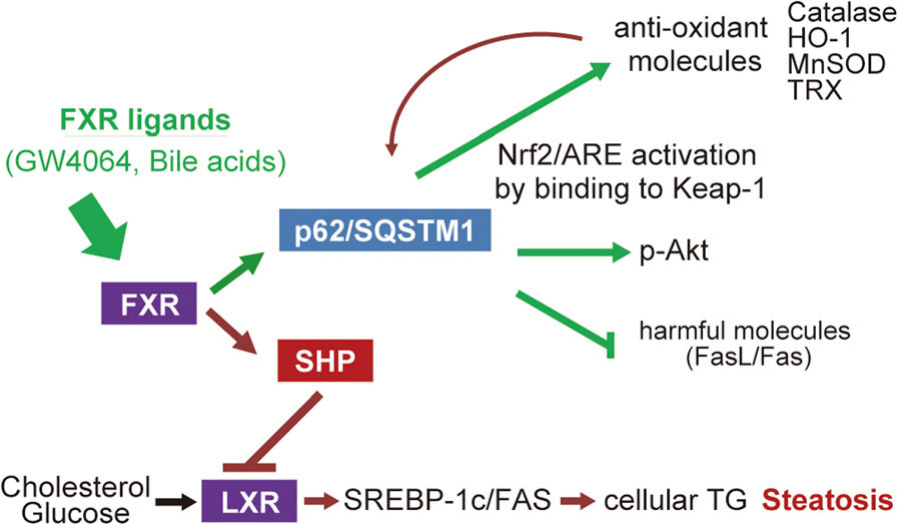
Schematic illustration of a crucial role of FXR-p62/SQSTM1 pathway in liver/hepatocyte protection. FXR upregulates Nrf2-regulated antioxidants, activates (phosphorylates) pro-survival Akt and suppresses harmful FasL/Fas via p62/SQSTM1, and therefore protects against liver/hepatocyte injury. FXR also improves steatosis of hepatocytes/liver possibly by suppressing LXR/SREBP-1c/FAS adipogenic pathway via SHP. FXR may be one of the key molecules in protecting steatotic hepatocyte/liver injury

By the way, FXR is a member of the nuclear receptor superfamily and is a ligand-activated transcription factor that is essential for maintaining mainly hepato-intestinal homeostasis [24, 25]. FXR has the effects against carcinogenesis and inflammation in liver and intestine as demonstrated by the development of inflammation and tumors of FXR knock-out mice [29]. However, the mechanisms of the physio-pathological effects of FXR are not completely understood. Recently, a novel target gene of FXR was identified in the regulation of *p62/SQSTM1* gene expression [29]. The anti-adipogenic effect of FXR also has been reported in hepatocytes [30]. FXR therefore has been a potent therapeutic target against NAFLD including NASH [28]. It is known that FXR upregulates SHP, which suppresses LXR/SREBP-1c/FAS-mediated production of TG in hepatocytes [27]. This promptly led us to the idea that upregulation of liver-protective p62/SQSTM1 by FXR may provide liver-specific protection against oxidative and Fas-mediated injuries. FXR-p62/SQSTM1 may send “protective signals” to steatotic hepatocytes and fatty liver which are vulnerable to oxidative stress and Fas-mediated death signals. Here in the present study, the treatment with GW4064 reduced TG contents in hepatocytes presumably by suppressing adipogenesis and promoting adipolysis (Fig. 4b and c). FXR suppressed accumulation of TG in AML12 cells possibly by reducing its cellular production through upregulating SHP and inhibiting the SREBP-1c/FAS pathway. Regarding adipolysis by LXR, further study has to be performed to confirm this result and elucidate the underlying mechanism. Though the treatment with FXR slightly lowered hepatic TG contents in db/db mice, the defatting effect of FXR was not evident in vivo. Because the model mice used in this study were diabetic with severe fatty liver (Fig. 5c), they might have been too extreme to evaluate correctly the effect of GW4064. In order to confirm the anti-adipogenic and/or adipolytic effects of FXR on liver steatosis, we must perform additional experiments by using the other physio-pathological mouse models. However, these properties of FXR indicate the potent contribution to the protection against fatty and even non-fatty livers from surgical stress and/or injury and to improve steatosis via p62/SQSTM1 and SHP in various clinical settings (Fig. 6).

Regarding the pro-survival and cell protective effects by FXR in hepatocytes, FXR-agonist showed mild but certain pro-survival and protective effects via p62/SQSTM1 in non-healthy steatotic hepatocytes as well as healthy hepatocytes (Figs. 3 and 4c), indicating the potent protective effect in various physio-pathological conditions of liver and intestine cells. These effects were confirmed by using the mouse surgical model with fatty liver. Post-PH liver injury was significantly improved by the treatment with LXR-agonist (Fig. 5a, b). Unfortunately, it failed to induce post-PH mitotic response (hepatocyte proliferation) and to regenerate the remnant liver significantly. The failure to promote post-PH mitotic response and regeneration may be explained by the negative effects of GW4064 on mitosis-associated proteins (Fig. 2e).

Whereas, Huang, W. et al. [31] and Xie, Y. et al. [32] reported the potential effects of FXR on hepatocyte proliferation in vitro and in vivo. FXR stimulated mitotic response in the post-PH mouse liver [31] and acetaminophen-treated mouse liver [32]. Regarding the in vitro experiments, Xie, Y. et al. used human cancer cell line (HepG2) which possesses autonomic proliferative capability, and we differently used AML12 cells in the present study, which were maintained by TGF-α secreted in autocrine/paracrine fashions. They studied MTT assay for evaluation of live cells and BrdU incorporation for proliferation in HepG2 cells. The live cell numbers and BrdU incorporation of HepG2 cells were certainly increased by the treatment with GW4064 (1.0–5.0 μM). In the paper, they concluded that FXR may promote proliferation of HepG2 tumor cells by activating pyruvate dehydrogenate kinse4 (PDK4)-mediated metabolic reprogramming and generating glycolytic intermediates required for cell proliferation, which was not observed in healthy hepatocytes. This indicates that FXR-stimulus may promote cell proliferation, but not be a sufficient condition for non-tumorous cells. The enhancement of proliferation by GW4064 may require basically the autonomic proliferative capability of cells (such as HepG2 cells). Taken together, we think that the hepatocytes with autonomic/strong proliferative capability can be stimulated with some kinds of trigger such as “metabolic switch”, whereas that of healthy hepatocytes whose proliferation is not driven autonomically/strongly cannot be stimulated by the metabolic changes alone. Similar to FXR, the association of p62/SQSTM1 with tumor have been reported [30, 33]. The anti-oxidant and pro-survival properties of p62/SQSTM1, is surely expected to prevent oxidative stress, cell death (injury) and inflammation in normal hepatocytes/liver. However, p62/SQSTM1, if accumulated excessively in cells, may contribute to oncogenesis, especially in liver.

These complicated mechanisms in FXR- and p62/SQSTM1-associated physio-pathology make it difficult to understand its role in various conditions. Therefore, it seems to be a limitation for interpretation of the physio-pathological roles of FXR and p62/SQSTM1 in the present study. In order to evaluate properly the proliferative/pro-survival and the other effects of FXR on liver physio-pathology, we must perform additional experiments using other ligands with various concentrations in healthy and non-healthy hepatocytes, and also confirm by using the other physio-pathological models.

## Conclusions

This study is the first to show the critical roles of the FXR-p62/SQSTM1 pathway in the protection against post-PH liver injury and possibly the FXR-SHP pathway in the improvement of liver steatosis. Further studies are required to elucidate thoroughly the mechanisms of protection against injury in steatotic and non-steatotic liver. However, the present data provide important clues toward the development of new therapies specifically against liver injury without affecting other organs/tissues.

## Additional files

Additional file 1 The nuclear translocation of Nrf2 by GW4064 (0, 0.5, 1.0 and 2.0 μM) was studied immunocytochemically in AML12 mouse liver cells. (PPTX 2650 kb)
Additional file 2 Liver X Receptor (LXR)-agonist did not affect serum levels of glucose (GLU) and triglyceride (TG) after PH in db/db mice with fatty liver. Serum levels of glucose and TG were not affected post-PH 24 and 72 h by the pre-treatment of GW4064 in db/db mice with fatty liver (5 mg/kg BW, refer to Materials and Methods in details). (PPTX 74 kb)

## Abbreviations

ALT: Alanine aminotransferase
AML12: Alpha mouse liver 12
ARE: Transcriptional activity on antioxidant response elements
AST: Aspartate aminotransferase
ATP: Abnormalities and decreased adenosine triphosphate
BSA: Bovine serum albumin
DMEM/F12: Dulbecco’s modified eagle medium: nutrient mixture F-12
FAS: Fatty acid synthase
FasL: Fas ligand
FXR: Farnesoid X receptor
H & E: Hematoxylin and eosin
HO-1: Heme oxygenase-1
HRP: Horseradish peroxidase
Keap-1: Kelch like ECH-associated protein-1
LDH: Lactate dehydrogenase
LXR: Liver X receptor
Mn-SOD: Manganese-dependent superoxide dismutase
NAFLD: Non-alcoholic fatty liver disease
NASH: Non-alcoholic steatohepatitis
Nrf2: Nuclear factor erythroid 2-related factor-2
OS: Oxidative stress
PBS: Phosphate buffered saline
PCNA: Proliferating cell nuclear antigen
PH: Partial hepatectomy
PVDF: Polyvinylidene difluoride
RFU: Relative fluorescence units
SDS-PAGE: Sodium dodecyl sulfate-poly acrylamide gel electrophoresis
SEM: Standard error of the mean
SHP: Small heterodimer partners
siRNAs: Small interfering RNAs
SREBP: Sterol regulatory element-binding protein
STAT3: Signal transducer and activator of transcription 3
TG: Triglyceride
TGF-α: Transforming growth factor-α
TRX: Thioredoxin
