# Supplementary material for: Relevance of FXR-p62/SQSTM1 pathway for survival and protection of mouse hepatocytes and liver, especially with steatosis
Source: BMC Gastroenterol. 2017 Jan 13;17:9. doi: 10.1186/s12876-016-0568-3 (PMC5237313; doi:10.1186/s12876-016-0568-3)
Supplement: Additional file 1 — The nuclear translocation of Nrf2 by GW4064 (0, 0.5, 1.0 and 2.0 μM) was studied immunocytochemically in AML12 mouse liver cells. (PPTX 2650 kb) [file 12876_2016_568_MOESM1_ESM.pptx]

## Slide 1
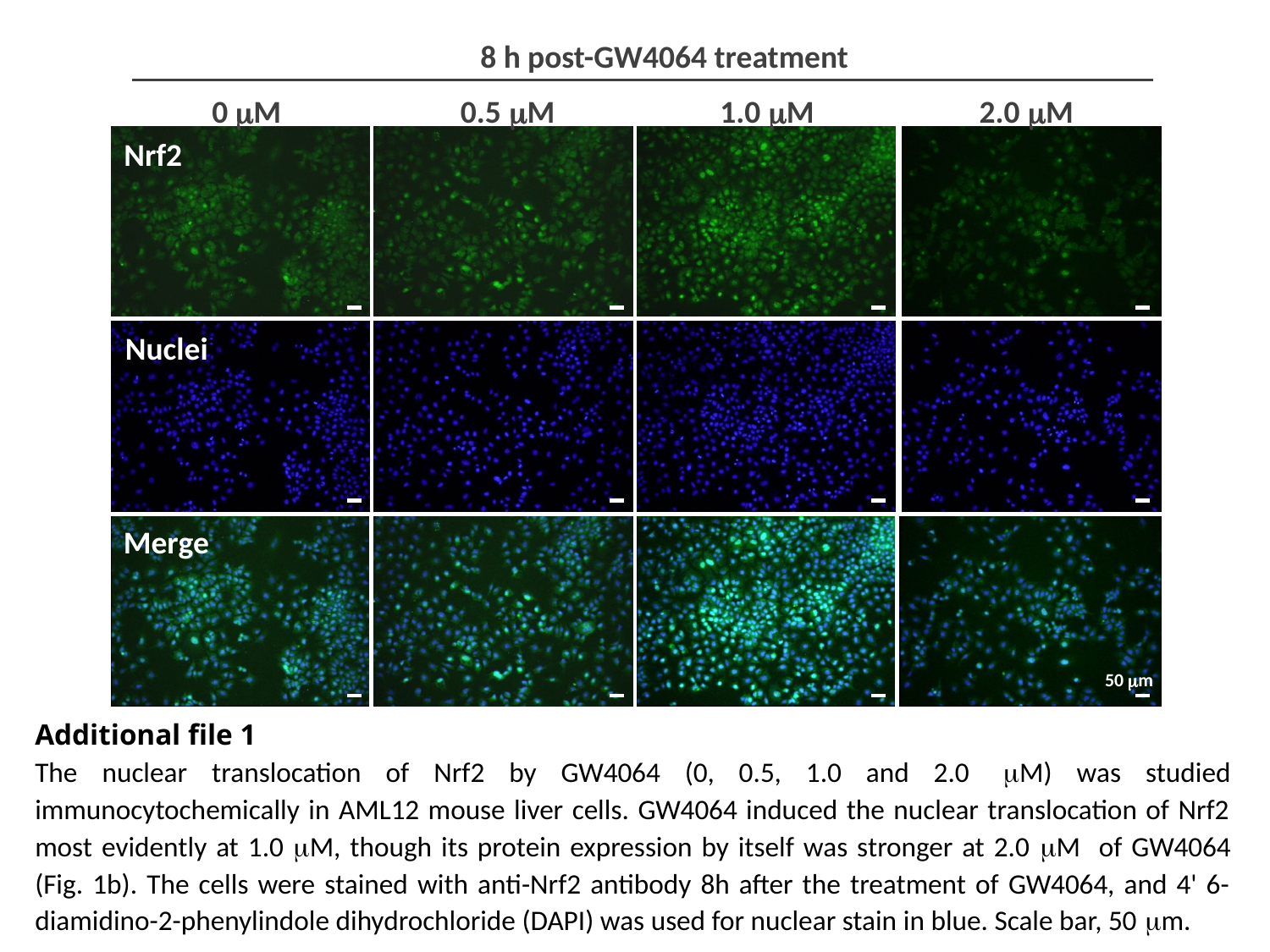

8 h post-GW4064 treatment
0 mM 0.5 mM 1.0 mM 2.0 mM
Nrf2
Nuclei
Merge
50 mm
Additional file 1
The nuclear translocation of Nrf2 by GW4064 (0, 0.5, 1.0 and 2.0 mM) was studied immunocytochemically in AML12 mouse liver cells. GW4064 induced the nuclear translocation of Nrf2 most evidently at 1.0 mM, though its protein expression by itself was stronger at 2.0 mM of GW4064 (Fig. 1b). The cells were stained with anti-Nrf2 antibody 8h after the treatment of GW4064, and 4' 6-diamidino-2-phenylindole dihydrochloride (DAPI) was used for nuclear stain in blue. Scale bar, 50 mm.
