# Supplementary material for: Relevance of FXR-p62/SQSTM1 pathway for survival and protection of mouse hepatocytes and liver, especially with steatosis
Source: BMC Gastroenterol. 2017 Jan 13;17:9. doi: 10.1186/s12876-016-0568-3 (PMC5237313; doi:10.1186/s12876-016-0568-3)
Supplement: Additional file 2 — Liver X Receptor (LXR)-agonist did not affect serum levels of glucose (GLU) and triglyceride (TG) after PH in db/db mice with fatty liver. Serum levels of glucose and TG were not affected post-PH 24 and 72 h by the pre-treatment of GW4064 in db/db mice with fatty liver (5 mg/kg BW, refer to Materials and Methods in details). (PPTX 74 kb) [file 12876_2016_568_MOESM2_ESM.pptx]

## Slide 1
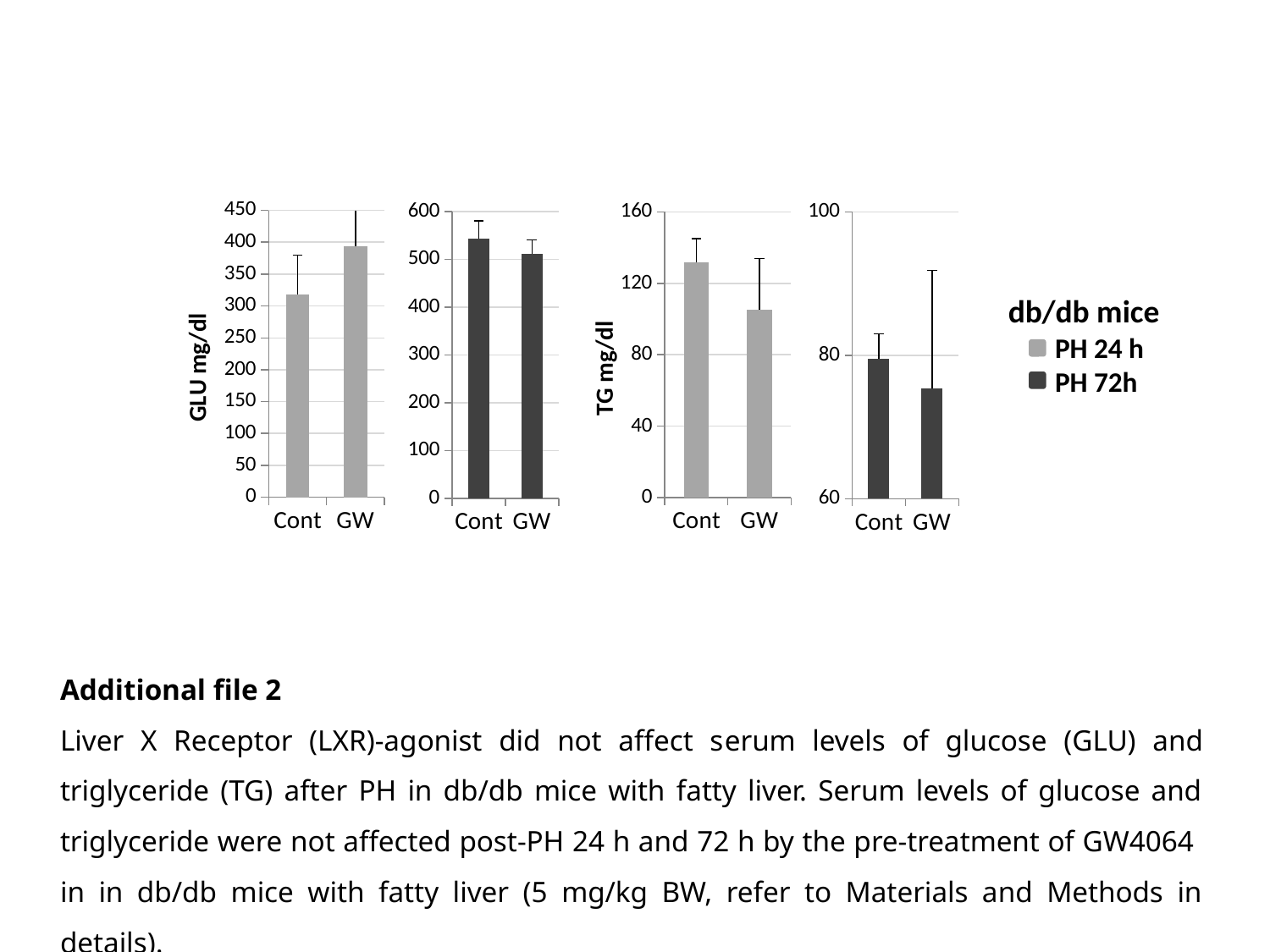

### Chart
| Category | |
|---|---|
| Cont | 542.5 |
| GW | 511.6666666666667 |
### Chart
| Category | |
|---|---|
| Cont | 318.3333333333333 |
| GW | 394.0 |
### Chart
| Category | |
|---|---|
| Cont | 131.66666666666666 |
| GW | 105.0 |
### Chart
| Category | |
|---|---|
| Cont | 79.5 |
| GW | 75.33333333333333 |db/db mice
PH 24 h
PH 72h
Additional file 2
Liver X Receptor (LXR)-agonist did not affect serum levels of glucose (GLU) and triglyceride (TG) after PH in db/db mice with fatty liver. Serum levels of glucose and triglyceride were not affected post-PH 24 h and 72 h by the pre-treatment of GW4064 in in db/db mice with fatty liver (5 mg/kg BW, refer to Materials and Methods in details).
